# “Having more women humanitarian leaders will help transform the humanitarian system”: challenges and opportunities for women leaders in conflict and humanitarian health

**DOI:** 10.1186/s13031-020-00330-9

**Published:** 2020-12-02

**Authors:** Preeti Patel, Kristen Meagher, Nassim El Achi, Abdulkarim Ekzayez, Richard Sullivan, Gemma Bowsher

**Affiliations:** 1 Department of War Studies, Conflict and Health Research Group, and R4HC-MENA, King’s College London, London, UK; 2 Research Associate, R4HC-MENA and Conflict and Health Research Group, King’s College London, London, UK; 3 Research Associate, R4HC-MENA, Global Health Institute, American University of Beirut, Beirut, Lebanon; 4 Professor of Cancer and Global Health, King’s College London, London, UK; 5 Senior Research Associate, Conflict and Health Research Group, King’s College London, London, UK

**Keywords:** Women, Leaders, Diversity, Inclusion, Health, Conflict, Humanitarian

## Abstract

**Background:**

It is estimated that over 40% of the half a million humanitarian workers who provide frontline care during emergencies, wars and disasters, are women. Women are at the forefront of improving health for conflict-affected populations through service delivery, education and capacity strengthening, advocacy and research. Women are also disproportionately affected by conflict and humanitarian emergencies. The growing evidence base demonstrating excess female morbidity and mortality reflects the necessity of evaluating the role of women in leadership driving health research, policy and programmatic interventions in conflict-related humanitarian contexts. Despite global commitments to improving gender equality, the issue of women leaders in conflict and humanitarian health has been given little or no attention. The aim of this paper focuses on three domains: importance, barriers and opportunities for women leaders in conflict and humanitarian health. Following thematic analysis of the material collected, we discuss the following themes: barriers of women’s leadership domain at societal level, and organisational level, which is subcategorized into culture and strategy. Building on the available opportunities and initiatives and on inspirational experiences of the limited number of women leaders in this field, recommendations for empowering and supporting women’s leadership in conflict health are presented.

**Methods:**

A desk-based literature review of academic and grey sources was conducted followed by thematic analysis.

**Results:**

There is very limited evidence on women leaders in conflict and humanitarian health. Some data shows that women have leadership skills that help to support more inclusive solutions which are incredibly important in this sector. However, deeply imbedded discrimination against women at the organisational, cultural, social, financial and political levels is exacerbated in conflict which makes it more challenging for women to progress in such settings.

**Conclusion:**

Advocating for women leaders in conflict and health in the humanitarian sector, governmental bodies, academia and the global health community is crucial to increasing effective interventions that adequately address the complexity and diversity of humanitarian crises.

## Key messages

- More women leaders can transform the humanitarian system to better meet the health needs of those affected by conflict and humanitarian emergencies.
- Women are significantly under-represented in the most senior humanitarian leadership roles.
- Patriarchal attitudes restrict women’s aspiration to becoming leaders in several conflict-affected societies.
- Humanitarian leadership teams that are more diverse and inclusive perform better.
- Organisational culture across the conflict and humanitarian health domain significantly hinders women pursuing leadership roles.
- Inclusive environments to enable women to become leaders creates broader understanding of the global health system.

## Introduction

The World Humanitarian Day on 19 August 2019 was dedicated to women, reflecting the growing global momentum on gender equality and equity. Marking the tenth anniversary of the occasion, the United Nations (UN) and several leading humanitarian organisations honoured the contribution of women humanitarian aid workers who provide life-saving support to millions of people caught in crises in some of the world’s most dangerous places [1]. It is estimated that over 40% of the half a million humanitarian workers, who provide frontline care during emergencies, wars and disasters, are women [2]. Women are also increasingly at the forefront of improving health for conflict-affected populations through service delivery, education and capacity strengthening, advocacy, and research [3, 4]. They have made important contributions to strengthening health systems, improving evidence and humanitarian interventions as well as in documenting human rights abuses, highlighting the detrimental health outcomes of marginalized groups, and bringing vital knowledge and intervention gaps to global attention [5].

At the same time, women are disproportionately affected by armed conflict and humanitarian emergencies [6]. In peacetime, women generally live longer than men, yet during armed conflict the gap between female and male life expectancy decreases as women, on average, suffer more from the indirect and long-term consequences of armed conflict, such as sexual violence, lack of access to healthcare and other socio-economic challenges [7, 8]. It is well documented that during conflict, women are more likely to experience intimate partner violence, as well as become victims of sexual violence as this is often used as a weapon of war [9–11]. Conflict itself promotes conditions during which existing gender inequalities and inequities are amplified; community structures, access to healthcare and human rights are all compromised resulting in worsening conditions for women [12]. Poverty and forced displacement are immediate secondary consequences of conflict, which expose women to further risk and vulnerability. Flight from violence places women at disproportionate risk of abduction, trafficking and sexual exploitation [13]. Once inside camps for displaced populations, women are at heightened risk of experiencing interpersonal violence, or further sexual exploitation in exchange for safety, or commodities such as food, water and shelter [14]. Health consequences of conflict again disproportionately fall along gendered lines resulting in high rates of maternal and neonatal mortality, and sexually transmitted infections. Over 60% of all otherwise preventable maternal deaths, 53% of all the world’s under-five deaths, and 45% of neonatal deaths occur in countries affected by humanitarian crises and fragile socio-political conditions where forced migration is also common [15–17]. This growing evidence base demonstrating excess female morbidity and mortality reflects the necessity of evaluating the role of women in leadership driving health research, policy and programmatic interventions in conflict-related humanitarian contexts.

In 2000, a landmark United Nations Security Council Resolution (UNSC 1325) on Women, Peace and Security, demanded greater representation and participation of women at all societal levels [18]. More recently, the World Humanitarian Summit in Istanbul in May 2016, the first Women Leaders in Global Health (WLGH) conference at Stanford University in October 2017, and follow-up conferences in November 2018 and 2019 on the same theme at the London School of Hygiene and Tropical Medicine and the University of Global Equity in Rwanda, addressed the significant barriers faced by women in global health, including in the conflict and humanitarian sphere. The WLGH events included key messages from leading female humanitarians and policymakers such as Dr. Joanne Liu, former President of Médecins Sans Frontières (MSF) and Dr. Soumya Swaminathan, Chief Scientist at the World Health Organisation (WHO) [19]. The idea that global health is a patriarchal system was powerfully emphasised by Dr. Swaminathan. This, combined with the entrenched unconscious bias towards women in leadership, is a significant barrier for women in all sectors. Joanne Liu stated that by creating inclusive environments to enable women to become leaders, we in turn create a broader understanding of the global health system [19].

In an era of protracted conflicts, the role and contribution of women within the conflict and humanitarian health domain has become increasingly important to understand and subsequently address the divergent needs of conflict-affected populations. However, women’s leadership within this domain has not attracted the same high-level attention as that of global health more broadly [20, 21]. The authors of this paper are invested in the evidence-led development of an agenda, established by the Women Leaders in Health and Conflict Initiative (WLHC), aimed specifically at remedying these longstanding inequities to realise the promise of advancing women’s leadership. Officially launched in October 2019, WLHC reflects the growing grass-roots momentum for such an initiative. It is inspired by broader global impetus to redress the significant gender-related barriers to leadership in global health and specifically for women in the conflict and humanitarian health domain [22].

This non-systematic exploratory review discusses core themes identified in the emerging literature on this topic by analysing underlying causes of persistent gender gaps that hinder women’s leadership in conflict and humanitarian health. It also aims to set the momentum for collecting evidence by providing recommendations for further research to influence policies affecting women leaders in conflict and health, both in the field and in academia, foster supportive work environments, and enable those working in the sector to realise the indispensable benefits of organisational diversity. To the best of our knowledge, this is the first review that focuses on exploring the barriers and facilitators for promoting women leadership in conflict and humanitarian health and the related research sector (Table 1).

**Table 1:** Key Terms and Definitions

**Gender:** Socially constructed roles, behaviours, activities and attributes that a given society considers appropriate for males and females. The term does not replace the biological definition of “sex,” nor the terms “women” and “men,” but emphasizes the existence of societal inequalities and stereotypes [23, 24].	
**Gender equality:** Individuals that identify with different genders are treated equally and ensuring that they have the same rights, opportunities and responsibilities, equal access to public goods and services, and equal outcomes [25, 26].	
**Gender equity:** Fairness and justice in the distribution of benefits and responsibilities between women and men, according to their respective needs. It is considered part of the process of achieving gender equality in terms of rights, benefits, obligations and opportunities [26, 27].	
**Gender pay gap:** The difference in the average hourly wage of all women and men across a workforce, as monitored by the Sustainable Development Goal indicator 8.5.1. The gender pay gap is not the same as unequal pay which is paying men and women differently for performing the same (or similar) work [20].	
**Conflict and Health:** Public health impact and responses related to armed conflict and humanitarian crises [28].	
**Diversity**: This includes differences according to gender, age, disability, race, cultural background, sexual orientation, social and economic background, profession, education, work experiences and organisational role [29, 30].	
**Organisational Culture:** shared understandings of the world, of the place of the organisation in the world, and of ‘normal’ behaviour around power, diversity and use of time; shared ways of thinking, feeling and understanding and the subsequent impact on the behaviours of individuals within an organisation, resulting in a collective culture [31].	
**Humanitarian leadership:** Leaders of humanitarian organisations who provide a clear vision and objectives for humanitarian action (whether at the program, organisational or system-wide level) [30, 32].	
**Motherhood leadership penalty:** Mothers with low participation rates in managerial positions [8].	
**Patriarchy:** The structural and ideological system that perpetuates the privileging of hegemonic masculinities. It is a hegemonic system of power relations based on gender norms that establishes the expected roles of men and women. In this system, women and girls have historically, and overwhelmingly, been oppressed, exploited or otherwise disadvantaged. So too have groups who do not conform with gender norms, the predominant binary approach to gender and sexuality, and/or heteronormative expectations. These include lesbian, gay, bisexual, transgender and intersex (LGBTI) populations, as well as certain groups of men and boys [33].	

## Methods

The review’s aim is to identify key themes within the existing literature on women’s leadership in the conflict and health domain in order to establish: 1) key barriers and opportunities for women in this sphere; 2) identify gaps in research and practice meriting further attention; 3) to consider how these issues might be remedied through the formulation of recommendations based on the review findings. This study is based on an exploratory desk-based non-systematic review of academic literature from PubMed, Scopus and WorldCat. Further relevant literature was obtained by extensively screening reference lists from key articles. An extensive review of grey sources was carried out since initial searches of Google showed that several recent reports relating to women’s leadership in the humanitarian and conflict sector are published by humanitarian agencies, development non-governmental organizations (NGOs) and gender activists. The main grey sources included Reliefweb and Devex. Further searched websites included those of leading international humanitarian agencies: Médecins Sans Frontières (MSF), International Committee of the Red Cross (ICRC), International Rescue Committee (IRC), Save the Children, Action Aid, Care International, Islamic Aid, Oxfam, World Vision, Islamic Relief. Other sources were found from global organisations including the World Bank, the International Monetary Fund, the World Economic Forum, Women Leaders in Global Health (WLGH), Overseas Development Institute (ODI), ALNAP, and relevant United Nations agencies (UNHCR, WHO, UNICEF, UNFPA, ILO and OCHA). Humanitarian and conflict specialist websites were included such as the Humanitarian Advisory Group, the Humanitarian Women’s Network, Humanitarian Practice Network (HPN), as well as conference reports from the Women Leaders in Global Health Conference 2018 and 2019 and the Lancet Series on advancing women in science, medicine and global health (February 2019). Online newspaper sources were also searched, including the Guardian and Reuters. Podcast information was reviewed from the Devprowomen 2030 audio series [34]. Material from social media sources such as Twitter and YouTube were included in the search. We created a WLHC twitter handle and through this process we searched for similar twitter handles to identify material that was relevant to our main objectives. We also followed the threads of specific global events, including World Humanitarian Day 2019 where women’s leadership in the humanitarian sector was discussed. Through snowballing we were able to track down reports, quotes and press materials which were publicised through social media. The Twitter accounts of the organisations mentioned in this review were the focus of the social media search and leading individuals working within those organisations. We did not, however, systematically search social media sources as this was an exploratory study and therefore a separate follow-up study specifically utilising social media as a core methodology would be useful.

For the inclusion criteria, English and Arabic search terms were used to source the material for this study. Search terms included: women’s leadership, women leaders, gender and leadership, female, health, humanitarian, humanitarianism, medical humanitarianism, development, conflict, war, NGO, civil society organisations, research, universities, think tanks, sexual harassment and humanitarian sector, organisational culture, humanitarian sector, motherhood, parenthood. The search excluded women’s leadership within clinical settings in humanitarian settings and women’s leadership within individual clinical outcomes (such as cancer, diabetes, infectious diseases, surgery) in humanitarian settings, military leadership in the conflict and humanitarian sphere. We felt these are very specialist domains that required separate studies. Our search also excluded other main languages contained in the main databases we searched. The timeframe for the search focused on contemporary conflict settings from 2000 to reflect UNSC 1325 which calls for greater participation and representation of women in situations of armed conflict. Our end date was September 2020.

The review protocol was divided into five stages: 1) searching for abstracts and other reports based on key-word search terminology; 2) selecting references for detailed reading; 3) peer-validation of reference inclusion 4) identifying recurring themes from selected references; 5) and aggregating these themes. PP, KM, NEA and GB were involved in all five stages of the review protocol.

Thematic analysis and interpretation of evidence from published and grey literature were analysed according to the process described by Braun and Clarke for thematic analysis of qualitative research which includes open, axial and selective coding [35, 36]. Articles were manually categorised in two phases, initially using the described open coding process to generate early themes and categories. Subsequently, emerging themes were synthesised before undergoing peer-verification between the authors. Following thematic analysis emerging themes were identified and categorised according to broader domains of ‘importance’, ‘barriers’ and ‘opportunities’. Within the barriers of women’s leadership theme, we categorised the findings into two subthemes: societal level, organisational level, which is subcategorized into culture and strategy. Finally, following identification and categorisation of themes, the authors developed a set of recommendations relating to the themes which underwent multiple rounds of revision and validation.

## Results

Overall, very limited evidence was found on women leaders in the field of conflict and humanitarian health [37]. Detailed results on the characteristics of selected studies can be found in Additional file 1. We screened just over 160 papers and reports and found 54 relevant ones which were reviewed and critically discussed. Of these 25 were from grey literature sources from humanitarian and development organisations, eleven peer reviewed articles, six from online news sources, four opinion pieces, three commentaries, three from conference materials, one press release, and one from social media. Only 19 of the sources included overall specifically discuss women leaders in this domain. Of these six were from grey literature sources from humanitarian and development organisations, three from online media sources, three peer reviewed articles, two from conference materials, two opinion pieces, one commentary, one press release and one from social media. The humanitarian and development organisations represented in the grey literature sources are: UNOCHA, WHO, ALNAP, Humanitarian Advisory Group, CARE, Centre for Humanitarian Leadership, ActionAid, OECD and Grand Challenges Canada. There are also limited data on the percentage representations of men and women at different levels of management and leadership in most humanitarian (and related) organisations. The majority of the sources discussed humanitarian and conflict settings in general without specifying exact locations, however, some sources specifically mention the following regions and countries including: Asia, Middle East and North Africa, Africa, Syria, Jordan, Yemen, Bangladesh, Nepal, Malawi, the Democratic Republic of Congo, and the Philippines.

We begin by discussing why women leaders in conflict and humanitarian health are important, then focus on key barriers that hinder women’s leadership in this domain, namely, societal, organisational culture, funding, and policy and practice, and finally highlight the available opportunities for WLHC.

## Role of women’s leadership

Studies on gender and leadership suggest that there are gendered differences in leadership styles; women tend to have a different yet complementary leadership style to men due to skills and strategies learnt whilst overcoming systemic barriers during their long stay in the mid-career phase, and capitalising on traits that they are traditionally associated with, including a more democratic and transformational approach to leadership than male counterparts [38–40]. A survey conducted by United Nations Office for the Coordination of Humanitarian Affairs (UN OCHA) of over 1000 women humanitarians in 115 countries found three unique attributes women bring to humanitarian action: firstly, the ability to speak to women from affected communities; secondly, unique perspectives; and lastly, a unique style of leadership [41].

Women’s representation and engagement in leadership roles would put women’s issues at the front of the global agenda, challenge the traditional hierarchies of knowledge and power by highlighting undervalued and unrecognised knowledge, and advocate for more inclusive, diverse and representative decisions [42, 43]. Therefore, rebalancing unequal power in the workplace by having more women leaders would improve organisational performance and revenues. Evidence from sectors such as the development, financial and private sectors show that greater diversity and inclusion results in macroeconomic growth, efficiency and better regulation overall [44, 45]. Christine Lagarde, current President of the European Central Bank and former President of the International Monetary Fund, argues that “employing more women and tackling sexism in the workplace is the key to making the world economy richer, more equal and less prone to financial collapses” [46]. The Global Gender Gap Report 2020 states that persistent gender inequality in leadership is a significant global economic risk and obstacle for human development. The average human development index for women is 6 % lower than that of men, with countries in the low development category suffering the widest gaps – which tend also to be countries impacted by conflict and humanitarian crises [47].

Some data suggest that leaders in this sector also require diverse, adaptable skillsets that enable them to effectively work across many cultures and contexts, working with communities, national and international staff and partners, whilst ensuring their leadership style facilitates capacity strengthening opportunities for national staff and partners [37, 48]. Evidence from leading humanitarian organisations suggests that global efforts to protect and assist people caught up in conflict and national disasters will be more effective if more women contribute in leadership roles [3, 49, 50]. In an article in the Lancet series on humanitarian health, Paul Spiegel states that the humanitarian system requires a major reform of leadership and coordination models, as the current system does not reflect the complexity and diversity of current humanitarian emergencies [51]. Spiegel further notes that for any leadership revision to occur, governments, UN agencies, multilateral organisations, and international NGOs “need to put aside differences and relinquish authority, influence, and funding.” A similar sentiment was reiterated by Lan Mercado, Asia Regional Director of Oxfam who recently stated that “having more women humanitarian leaders will help transform the humanitarian system” [52]. This may in turn, assist in the leadership change required to better meet the needs of those affected by conflict and humanitarian emergencies.

Indeed, Cooperative for Assistance and Relief Everywhere (CARE) International’s research on women working in the humanitarian sector shows that women might be better at identifying needs and realities of different groups; they may be able to use social capital and networks to reach other women at different geographical levels; they might provide a space for women’s voices, and supporting women’s leadership potential; provide solidarity to other women and girls in day-to-day spaces and activism; and may help to make interventions gender transformative, and potentially more sustainable [53]. However, unlocking the benefits of greater diversity in the conflict and health domain requires focused action to address the underlying causes of persistent gender gaps in a systemic way.

## Barriers hindering women’s leadership in conflict and humanitarian health

Although women’s leadership in global health has attracted high-level attention, there are major challenges in addressing women’s leadership, representation and participation in the conflict and humanitarian health domain. There is a visible gap in advancing women’s leadership at this nexus, as their work often falls outside the margins of global health [54, 55]. A peripheral subject in global health, the domain of conflict and humanitarian health also uniquely positions itself in non-permissive and often highly securitised and politicised contexts in which attitudes and assumptions towards leadership are predominantly male-normed [56–58]. Most of the literature that discusses gender inequality focuses on the various barriers that women face in order to join the job market and/or excel in their careers to reach managerial positions in various settings including the humanitarian sector. Those barriers are deeply entrenched at the societal and organisational levels.

### Societal level

Entrenched socio-cultural gender discrimination results in an immense pool of untapped talent in many countries. Patriarchal sociocultural values and their associated gender ideologies are negatively related to women’s career development, limiting career choices to those that adhere to the traditional division of labour and which do not compromise domestic responsibilities [4, 59]. Women are also expected to occupy lower-level roles due to culturally coded linkages between leadership and masculinity. For example, women remain largely underrepresented at all levels of governance in the Arab region with the average proportion of female members of parliament is 19%, which is below the global average (25%) [47, 60]. Even in “women friendly” sectors, a study conducted on women leadership in academia in the Arab world, found that women lead fewer than 7 % of Arab higher-education institutions (48 out of 702 universities) [61]. This non-permissive environment impacts women’s construction of their leadership identities and their self-perception as leaders, which results in low self-confidence and discouragement from the pursuit of high-level positions. The few who decide to break the norm could be subjected to discrimination, opposition, life threats, or imprisonment [38].

Many of these barriers are echoed in humanitarian, fragile and post-conflict contexts including, but not limited to, Bangladesh, Ethiopia, Nepal, Gaza, the Philippines, Sierra Leone, Zimbabwe, northern Uganda, Cambodia, Guatemala, El Salvador, Honduras and Nicaragua [4, 62, 63]. The barriers reported in these settings include socio-cultural and economic obstacles for women exercising agency and leadership in humanitarian crises; patriarchal attitudes and norms that restrict women’s participation in public space and undermine their contribution as leaders; women’s burden of unpaid work; a lack of experience and opportunities to participate in leadership, exclusion from emergency response decision-making structures; low self-confidence; poverty and access to resources; and low levels of education and literacy [4, 64].

### Organisational level

Organisational culture across the conflict and humanitarian health domain is a replication of societal level challenges as it is discriminatory, deeply misogynistic and generally hinders women pursuing leadership roles [63]. Few of the leading organisations working in this sphere have (or have had) a woman in their leading managerial role although Oxfam, MSF, and the WHO are notable exceptions, with Winnie Byanyima and Joanne Liu both being appointed to the top leadership roles at Oxfam and MSF respectively in 2013. Margaret Chan was WHO Director General from 2006 to 2017 and Gro Harlem Brundtland served in that role from 1998 to 2003. WHO is one of the few UN agencies to have had a female Director General; indeed the UN has never appointed a female Secretary General. Organisational cultures in conflict and humanitarian health and beyond tend to be gendered, meaning that assumptions about leaders and the contributors to effective leadership are typically male-normed [56–58].

Security issues can reduce women’s representation in humanitarian leadership. In extreme security risk countries, men tend to dominate leadership positions, filling between 60 and 69% of leadership positions. Interviewees from a study on diversity in humanitarian settings feel that high security risk contexts often lead to the exclusion of women [30]. Researchers from the Feinstein International Centre interviewed individuals from humanitarian and development organisations and found key operational factors conducive to sexual misconduct across the organisations: the majority of aid operations are led and dominated by men, especially in situations where active armed conflict is occurring; male domination of power and decision-making in aid agencies contributes to a macho environment, where males with power foster an atmosphere where sexual discrimination and harassment flourishes [65]. While CARE international and ActionAid have embedded gender equity approaches into their working cultures, an analysis of power structures at ActionAid and anecdotal evidence from CARE international have highlighted a lingering culture of a ‘boys club’ that still affects some leadership contexts [66]. One interviewee felt that men are given greater responsibilities even when they are not suitably qualified. Women disclosed being nervous about raising gender specific issues, for example security or maternal responsibilities, for fear of being perceived as ‘weak’ and not being offered leadership roles as a consequence [66].

In 2018, Oxfam and Save the Children were rocked by a cascade of sexual misconduct accusations. It was revealed that Oxfam UK staff, including the Country Director, had been paying local young women for sex in Haiti whilst working on the humanitarian response to the 2010 earthquake [16]. Following this, further allegations of sexual abuse and misconduct of staff within aid organisations emerged, including Save the Children, including allegations of poor standards of process and governance in the way some of these cases have been dealt with [16]. In addition to the reputational damage caused by sexual misconduct, harmful organisational cultures also have a strong impact on financing shortfalls. In light of the sexual exploitation scandal in Haiti within Oxfam, the organisation was forced to make £16 million cuts to aid projects with several private donors being cancelled [67], .while Save the Children was suspended from its bidding for UK government funding [68].

To mitigate these issues, zero tolerance policies of sexual harassment and gender-based violence have been implemented in a number of organisations working in conflict-affected areas. While this is a significant development, it is insufficient to stymie the prevalence of sexual misconduct. The UN adopted such a policy in 2018, after a survey found that one third of UN staff and contractors experienced sexual harassment [1]. Large aid organisations working across multiple countries and contexts have found that instilling a comprehensive zero tolerance culture can be challenging, particularly when contractors are not covered by their sexual harassment and abuse policies [69, 70]. Helen Clarke, former Head of the UN Development Programme, urges organisations to act on their promises of zero tolerance in the workplace in order to see more women take on leadership roles and improve gender parity [71]. In a recent report, Deloitte (a large multinational accounting company) states that strategies designed to address conduct and culture in the workplace fail upon implementation because they conflict with entrenched practices [72]. Furthermore, the report notes that organisations must implement reporting mechanisms that employees can utilise without fear of reprisals.

Evidence also shows that sexism, sexual harassment, gender pay inequity, and fewer chances for promotion are key barriers that women in the scientific, medical and academic sector face [48, 62, 73, 74]. Laurie Garrett’s article in the BMJ provides recent analysis of the barriers experienced by women leaders in the scientific and medical sector. Female advancement in this sector faces significant barriers in access to advanced education, career progression and promotions, extreme bias in research funding, access to journal publication, and invitations to present at high-level meetings [75]. Several factors have been identified: inadequate guidance and mentoring, difficulty balancing family responsibilities while meeting promotion criteria (especially mid-career), and overt bias and gender discrimination in the workplace [76]. People are also less likely to recognise leadership qualities in women than in men [77, 78].

At a recent research symposium in London on *Conducting Research in Complex Environments*, Dr. Aula Abbara drew attention to disparities in the experiences of female health researchers in Lebanon who are increasingly conducting the on-the-ground research whilst being excluded from the processes of recognition [72, 79]. A study specifically investigating gender-based challenges of female health trainees and professionals in research institutions found that many participants viewed gender discrimination as a normal part of their culture [48]. As for sexual harassment, most of the studies on harassment within academia are limited in sample size. Sexual harassment is underreported since many academic institutes lack a reliable and transparent reporting mechanism [80–82]. Other factors contributing to underreporting include stigma, especially in conservative societies, fears of job loss in a highly competitive market, and power imbalances [83].

Many organisations include gender equality and empowerment as part of their core missions in various settings. In 2016–2017, the total aid target focused on gender equality from the Organisation for Economic Co-operation and Development (OECD) was at its highest amount. However, support for aid programmes specifically dedicated to gender equality and women’s empowerment as their principal objective remained below 4 % (4.6 billion USD) of total bilateral allocable aid showing a major gap in funding such projects [84, 85]. A study also demonstrated a lack of long-term support for gender equality projects as countries receiving such funds in 2008 were found to be no longer among the top recipients in 2013 [86].

Besides funding constraints, which are very common for development projects, gender equality and empowerment projects face additional challenges in humanitarian settings. For instance, a local NGO working on women’s empowerment in Syria reported challenges in finding a balance between local needs and donor conditionality. For example, a donor’s pre-defined strategic priority was to fund an intervention focusing on women’s political participation while neglecting a highly needed psycho-social support scheme. Similarly, the same NGO struggled with a another donor that insisted on focusing exclusively on gender based violence (GBV) but through a counterterrorism lens [87].

Another study highlighted how gender empowerment projects in conflict settings are still being conducted, monitored and evaluated using the same purposive and extractive approaches which focus on numbers rather than needs. In other words, projects which are intended to be feminist and impactful are merely transactional. They are not conducted using feminist research designs, which incorporate reflexivity and reciprocity and challenge the intersecting power hierarchies that negatively affect women, as the current humanitarian system lacks diversity and inclusion [88]. The weakness of the humanitarian system in terms of inclusion and support of women leadership was described in a recent report on local NGOs led by women in Bangladesh and South Sudan. The report explored how the “dearth of examples of women’s leadership related specifically to local humanitarian leadership does not reflect a lack of women’s leadership in this context”, but rather local NGOs led by women were found to lack the required support and recognition in which their efforts are not recognised in the humanitarian system [89].

The prevalence of detrimental policy and practice, including gender pay inequity and the motherhood leadership penalty, has been demonstrated across a number of sectors; but it is not well documented in the conflict and humanitarian health domain. A recent International Labour Organisation report highlights the issue of the motherhood leadership penalty in which mothers of young children have the lowest participation rates in managerial and leadership positions: only 25.1% of managers with children under 6 years of age are women, and for women without young children, 31.4% are managers. Where men share unpaid care work more equally with women, more women are found in managerial positions [13]. Some humanitarian organisations, such as Islamic Relief, recognise the vital role of women in leadership and have introduced more transparent, flexible internal roster recruitment processes [66]. Embedded policies such as these would encourage more women to take up leadership roles, particularly in conflict and humanitarian health where the unique demands of a humanitarian career disadvantage those with caring responsibilities, typically mothers of children [5]. While many of the required changes to support working women require implementation at the national policy level, organisations can support mothers by implementing more flexible policies and working environments. Parenthood is a key determinant of equality of career opportunities for women and men in all sectors. Furthermore, redefining family-caring roles as shared rather than the principal responsibility of women promotes women’s retention and progression in the workforce [25].

## Opportunities for women leaders in conflict and health

Although the bulk of the literature highlights significant barriers to women’s leadership roles in the conflict and humanitarian health domain, there are also some key opportunities for women leaders in conflict and health. Action Aid’s field research shows that conflict and humanitarian crises create potential spaces to challenge the barriers: women’s rights advocacy and localised responses facilitate shifts in power and resources can transform gender relations and empower women over the longer term [64]. Similar trends in breaking the gender norms were also observed in the Middle East and North Africa (MENA) region [90]. Following the Arab Spring (I) in 2011 and Arab Spring (II) in 2019, women were at the frontline of protests, which has challenged the deeply-embedded institutional and cultural barriers to gender equity [91].

The Syrian crisis has also created opportunities for women in host countries such as Jordan and Lebanon to work in the humanitarian field. For example, with a highly educated female workforce and an overall low employment rate among women in Jordan (21%), the influx of international humanitarian organisations has provided new career opportunities for women especially as humanitarian work is seen as an extension of the traditional more female-dominated domains such as health, education and social work. Since most of the international NGOs provide vacancies of equal opportunities where people are recruited based on skills, women are more capable of fulfilling senior positions in the humanitarian field and of “silently” defying the odds by working in traditionally male-dominated professions within NGOs [92].

Southern perspectives on barriers and opportunities for women leaders in conflict and health within the broader Women in Global Health movement have been largely missing from the current dialogues at leading international conferences, academic outputs and other events. Conferences such as the 10th anniversary of Empowering Women in Science in Kuwait in October 2017, highlighted achievements, challenges, areas for further research and policy for women in the MENA region and globally [93]. Why women have been left behind in leadership in the Global South was a major theme at a conference on Accelerating Women’s Health Agenda: Priorities and Opportunities Through Sustainable Development Goals, in Kenya in November 2018. Around the same time, the Manila Declaration set out ambitious targets for 50% of all programmes to have women as leaders across the Red Cross Societies [49]. These events and targets will hopefully set a trend for other organisations to ensure more equitable pathways for several young women aspiring to become women leaders in conflict and health [3].

Creating strong networks is important as it provides a space for women working in a specific sector to learn from one another, support one another, and advocate for change. Nasra Ismail, Acting Director of the Somalia NGO Consortium and former Country Director of Oxfam Somalia, encourages the establishment of coalitions between women in the Global North and Global South to build on lessons learnt from the shift in policies and regulations that have empowered women leaders in the Global North [71, 94]. Enhanced collaboration between researchers and practitioners will strengthen networks. Academia is a powerful tool through which we can address issues of injustice and bring the voices of excluded people and minority groups to the forefront [95]. Having more women leaders as editors and on editorial boards of leading journals would support this [96, 97]. Improved collaboration can also enhance evidence-based research with knowledge from experts in the field to impact policies that support women leaders in conflict and health. It is therefore imperative to create a platform to build these coalitions and undertake research across a range of humanitarian institutions to work towards achieving gender parity in conflict and health. This will in turn influence systemic change in organisational culture, policy and practice, creating enabling environments for aspiring women leaders [98].

New funding streams and activities led by Research for Health in Humanitarian Crises (R2HC)-ELRHA, NIH Fogarty, Hope in Conflict, and others aim to encourage more research in humanitarian emergencies, increase collaboration among investigators and aid organizations, and identify strategies to ensure uptake of evidence into policy and practice [99]. About a third of proposals for “Creating Hope in Conflict: A Humanitarian Grand Challenge,” and about half of all successful research bids for R2HC came from women although it is unclear how many of these women were based in high, middle or low-income countries [100, 101].

Prominent organisations in the humanitarian health and conflict sector must lead the way. The UN is arguably the world’s leading humanitarian organisation, whose work impacts those working on conflict and health in development and humanitarian settings. It must therefore set a leading example, and not electing a female Secretary-General in 2016 was a missed opportunity. The UN has made progress on gender equality in leadership; gender parity was again met at top levels of UN leadership, yet women continue to hold fewer positions than men at middle-management level. A 2019 report shows that only about one third of the UN’s Humanitarian Coordinators are women [102]. A number of UN agencies, including United Nations High Commissioner for Refugees (UNHCR), UNOCHA, UN Women, have established platforms to promote the voices and experiences of women working in conflict and humanitarian emergencies [103]. The UN, therefore, is in an opportune position to drive gender-transformative change that encourages diverse leadership across sectors by acting on its commitments and engaging with a wide range of stakeholders.

## Discussion and recommendations

Whilst the underlying causes constraining women’s leadership in conflict and humanitarian health are comparable with global data across other sectors, and despite a growing appetite for evidence, there is a significant gap in the availability of data and research specific to the conflict and humanitarian health domain. This is a significant hindrance not only for aspiring women leaders, but for women and girls inadvertently affected by armed conflict. The key barriers, opportunities and recommendations that have been captured in this review are summarised in Table 2.

**Table 2:** Summary of Key Barriers, Opportunities and Recommendations for Women Leaders in Health and Conflict

Key Barriers	Opportunities	Recommendations
Societal [4, 59, 62, 63, 66]	Women’s rights advocacy and localised responses [64, 71, 93].	Community-based dialogs and trainings. Organised diffusion: Participant-led method to share information with non-participating members [104].
Organisational culture [56, 58]	Embedding gender equity approaches in working cultures [66].	Wide-ranging strategies and reforms that challenge deeply entrenched practices. All institutions involved in conflict and humanitarian action should also undertake gender audits of their organisational culture and human resource management and set milestones to increase female leadership and gender sensitivity at all levels. Training on gender sensitivity and unconscious bias must be ongoing and built into a wide-range of activities [50, 57].
Organisational policy and commitments [44]	Developing inclusive policies and commitments [44].	Incorporate a commitment to gender equality within programme policies, visions, mission statements and core strategies [3, 43, 44].
Sexual harassment and gender-based violence [62, 73, 74]	Improving reporting mechanisms [62].	Adopting zero tolerance policies on sexual harassment and gender-based violence. Hiring independent regulatory bodies to conduct investigations. Obtain data on sexual violence and gender differentiated analysis to better understand the risks and appropriate mitigation strategies [62, 73, 74].
Gender pay inequity [49]	Governmental intervention for transparent salary reporting [84, 85].	Tracking gender pay in the humanitarian sector. Skills-based hiring [85, 93].
Motherhood leadership penalty [30]	Introducing internal roster recruitment [66].	Implementing paternal leave, flexible work policies [25, 26, 92].
Mentorship, training and collaboration [22, 50, 58, 75]	The importance of facilitating networks to benefit women aspiring to leadership positions cannot be underestimated, nor can the importance of the women and men who are in leadership roles committing to support those women who aspire to do the same. North-South partnerships are furthermore important, as women leaders in the Global South face compounded burdens in fragile contexts that often marginalize women and exhibit significant regulatory and cultural barriers [94, 98].	Effective mentoring through encouraging, identifying, and generating opportunities for women at early and mid-career stages in order to develop their creative ideas and leadership potential. PhD funding for women in Low and Middle-Income Countries (LMICs). More women leaders to serve as editors and on editorial boards of leading journals. Nominating women leaders in conflict and health for prizes, visiting fellowships, honorary doctorates, and other awards [94, 97, 98].
Power and resources [105]	Women’s rights advocacy, localised responses [87, 89].	Formal leadership training for early and mid-career women in humanitarian and conflict-related health sectors. Grant writing academies for women researchers – especially via the small (to medium) grant schemes for early career women researchers and post-doctoral candidates [58].
Insufficient donor funding [106]	Presence of few schemes that address women empowerment [93, 107].	New funding streams and activities that address gender inequality at the local, regional and global scales [84, 106, 108].
Inadequate research [55, 99]	There is no data available on how much funding is allocated to women’s leadership in the humanitarian sector. Quantitative research needs to be conducted to create evidence which promotes targeted funding. An examination of gender as an important locus of inequity in health capacity research is necessary and the inclusion of female professionals in capacity strengthening programmes should be seen as essential [90, 98].	Feminist research design that seeks to challenge the intersecting power hierarchies that negatively affect women. Bibliometrics and mapping of author contributions published on conflict and humanitarian health. Mapping gender composition of leadership teams across a series of humanitarian responses (which does not currently exist) [30, 88, 109].

As the research suggests, attaining leadership positions is a serious challenge for women across numerous sectors and this is perhaps more pronounced in the humanitarian sector, and subsequently the conflict and humanitarian health domain. Conducting research, with feminist designs, to determine the leadership needs by interviewing a diverse sample of practitioners and academics is a vital next step in research on this issue [42].

Challenging patriarchy should be an essential component of principled humanitarian action [33]. Humanitarian action focused on promoting gender equality can be characterised as gender-transformative [33]. Examples of gender-transformative programming include women’s empowerment through livelihoods, or the promotion of women’s participation in decision-making processes [33]

The transformation of organisational culture necessitates more than policy change, it must encompass wide-ranging strategies and reform that challenges deeply entrenched practices. As noted earlier, for any leadership revision to occur, the multiplicity of actors and organisations involved “need to put aside differences and relinquish authority, influence, and funding.” We argue that one of the key reforms that need to be looked at is the gender gap in leadership and how to utilise women’s leadership skills in such a reform. Evidence suggests the lack of interventions to foster a supportive organisational culture that support women’s career pathways is often the result of women’s under-representation in management and leadership positions [2]. Given the unique nature and associated risks of women working in the conflict and humanitarian health domain, there is a growing urgency for such practices to be better understood, analysed and addressed through policy reform. Without such reform it will be difficult to achieve the change required to create enabling environments that support more women in leadership roles within the conflict and humanitarian health sector. However, it should be noted that the humanitarian field is highly unregulated, especially in conflicts, with local NGOs operating in silos without proper monitoring from independent regulators. Implementing these policy reforms to include all operating NGOs is thus important to achieve safer working environments for women in the humanitarian sector.

Beside policy reforms, a study from the Organisation for Economic Co-operation and Development- Development Assistance Committee (OECD–DAC) looked at how donors can improve the effectiveness of their support to gender equality and women’s rights in conflict situations: basing programmes on a more nuanced understanding of the links between gender, conflict, and fragility; adapting ways of working to rapidly changing fragile contexts; and strengthening donor coordination around gender, which requires looking beyond individual projects to address root causes of gender inequalities and achieve common goals [86]. Evidence from case studies in Nepal highlighted the considerable impact that strengthened organisational leadership on gender, accompanied by concrete incentives, can have on the quality of programming [108]. In the same study, the health sector behavioural change project reviewed in the Democratic Republic of Congo (DRC) focused on local patterns of Sexual and Gender Based Violence (SGBV) rather than the broader national conflict, even though some of the programme’s target areas were directly conflict-affected and the strong links between SGBV and the wider conflict context are widely recognised in the DRC. While it would be beyond the mandate and capacities of such SGBV programmes to seek to address national or regional-level conflicts, more work could be done to understand the links between different levels of conflict and violence and how programming on one level may contribute to positive or negative change on other levels [108]. Indeed, in addition to strong collaboration between actors, diverse leadership may support the strengthening of donor coordination on gender targeted programmes that intrinsically link conflict and humanitarian health.

While increasing the role of women’s leadership in conflict and humanitarian health is important, and as evidenced in this paper may have positive benefits, there are also possible disadvantages as raised in other sectors. Firstly, the male dominated environments of many conflict and humanitarian settings may not be conducive to female leadership in terms of taking direction from women leaders. This can be attributed to the incongruity of the traditional female role and expectations of women arising in male-dominated settings and organisations [110]. Secondly, increasing the number of women in leadership roles may create a “diversity paradox.” One study on political leadership demonstrated that the use of a quota system increased the number of qualified female candidates without increasing the diversity among women within the group. This diversity paradox may also inhibit the ability of individuals to work together effectively [111]. Most of the literature treats women homogenously, contributing the lack of women’s leadership in academia and the humanitarian and development sectors to embedded patriarchy and gendered norms. This brings into discussion the importance of intersectionality, for example race, socioeconomic status, and how this intersects with gender to create unique experiences of marginalization and disadvantage [112, 113]. Thirdly, negative intra-gender relations, including competition and ostracising other women, is prevalent across various sectors and can constrain and undermine women’s progress [114]. All of these areas merit further examination to improve our understanding of how diverse leadership can be implemented to counter leadership inequities that drive change and improve prospects for women working in conflict and humanitarian health.

Lastly, we recommend a systematic review as a next step for researchers interested in this topic to further understand the gaps in evidence, building on not only the role of women but also intersectionality, and how to advance diverse leadership in the conflict and humanitarian health domain.

### Limitations

Due to the novelty of the topic and to the scarcity in data related to women’s leadership in conflict and health in academic and grey literature, our efforts to identify relevant literature went beyond the usual efforts applied in similar narrative reviews, rendering the review to be non-systematic.

Another potential limitation of the review is that most of the information was from the grey literature and social media. However, we regard these sources to be valuable as they provide a broader overview of the topic and also offer a starting point for more systematic research.

A further limitation is that the search only included reports and journal articles that are written in English and Arabic and it is unknown if further insights are to be gathered from expanding the languages of contributing literature. For further interviewing work, this should be a consideration to access a diversity of voices and cultures.

## Conclusion

Women face a multitude of barriers to participation in leadership spheres in conflict and humanitarian health. Professional advancement is hindered across various stages of professional development in both the practitioner and research environments. Unsupportive organisational cultures, significant persistent motherhood leadership penalties and systemic obstacles to accessing opportunities for career progression continue to hamper women in this particularly male-dominated sphere. Practitioners suffer exclusion in the macho operational environments of conflict and humanitarian action, and recent sexual abuse scandals have further compounded the structural inequities and far-reaching damage caused by longstanding gender inequities in leadership and organisational culture. Women researchers in this field also experience discrimination at various tiers of professional life; accessing research funding, overcoming cultural barriers to research and receiving recognition for research activity which translates into career progression for women remains especially challenging in this sphere.

This paper has examined the nascent evidence base and sought to use emerging themes and ideas to develop a series of recommendations for supporting efforts to promote women in research. Research career pathway development, organisational culture change, increasing specific funding for women leaders, social media activities and enhancing networking and mentoring activities are critical domains for developing a professional field supportive of female advancement at the highest levels. Adopting a targeted approach with the explicit goal of remedying existing structures of patriarchy is overdue. The importance of generating research momentum to examine the existing disparities and effectiveness of programmes to counter leadership inequities is essential to drive change and improve prospects for women working in conflict and humanitarian health.

## Supplementary Information

**Additional file 1.**

## Data Availability

All data generated or analysed during this study are included in this published article.

## Abbreviations

CARE: Cooperative for Assistance and Relief Everywhere
DRC: Democratic Republic of Congo
GBV: Gender Based Violence
LGBTI: Lesbian, gay, bisexual, transgender and intersex
LMIC: Low and Middle-Income Country
MENA: Middle East and North Africa
MSF: Médecins Sans Frontières
NGO: Non-Governmental Organisation
OECD-DAC: Organisation for Economic Co-operation and Development- Development Assistance Committee
R2HC: Research for Health in Humanitarian Crises
SGBV: Sexual and Gender Based Violence
UN: United Nations
UNHCR: United Nations High Commissioner for Refugees
UNICEF: United Nations International Children’s Emergency Fund
UN OCHA: United Nations Office for the Coordination of Humanitarian Affairs
WHO: World Health Organization
WLHC: Women Leaders in Health and Conflict Initiative
WLGH: Women Leaders in Global Health
