# Supplementary material for: “Having more women humanitarian leaders will help transform the humanitarian system”: challenges and opportunities for women leaders in conflict and humanitarian health
Source: Confl Health. 2020 Dec 2;14:84. doi: 10.1186/s13031-020-00330-9 (PMC7709302; doi:10.1186/s13031-020-00330-9)
Supplement: Supplementary file 1 — Additional file 1. [file 13031_2020_330_MOESM1_ESM.docx]

| **Reference** | **Author(s)** | **Year** | **Organisation** | **Publication Type** | **Country/Population** | **Key focus on women’s leadership** |
| --- | --- | --- | --- | --- | --- | --- |
| 1 | Howard Mollett | 2017 | CARE | Grey | Jordan, Philippines | Women’s activism. |
| 2 | Sophie Witter, Justine Namakula, Haja Wurie^,^ Yotamu Chirwa^,^ Sovanarith So, Sreytouch Vong, Bandeth Ros, Stephen Buzuzi, Sally Theobald | 2017 |  | Published | Sierra Leone, Zimbabwe, northern Uganda and Cambodia | Gender relations in health workforce. |
| 3 | Reuters | 2019 | Reuters | Grey | None specified | Organisational culture and sexual harassment. |
| 4 | UNOCHA | 2019 | UNOCHA | Grey | Global humanitarian settings | Women humanitarians. |
| 5 | Julie Lafrenière, Caroline Sweetman, Theresia Thylin | 2019 |  | Published | Global humanitarian settings | Women’s empowerment and increasing recognition of women’s leadership roles in humanitarian settings. |
| 6 | UNOCHA | 2019 | UNOCHA | Grey | Global humanitarian settings | Not applicable |
| 7 | Thomas Plümper, Eric Neumayer | 2006 |  | Published | Global conflict settings | Disproportionality of effect of conflict on women. |
| 8 | Paul Clarke, Ben Ramalingam | 2008 | ALNAP | Grey | None specified | Organisational structure in humanitarian organisations. |
| 9 | Khuloud Alsaba, Anuj Kapilashrami | 2019 |  | Published | Syria | Not applicable |
| 10 | Jacqui Tree | 2012 |  | Published | None specified | Not applicable |
| 11 | Kirsten Johnson,Jennifer Scott,Bigy Rughita  Michael Kisielewski, Jana Asher, Ricardo Ong, Lynn Lawry | 2010 |  | Published | Eastern Democratic Republic of the Congo | Not applicable |
| 12 | Carol Cohn |  |  | Published | Global conflict settings | Disproportionality of conflict on women. |
| 13 | Rothkegel S, Poluda J, Wonani C, Papy J, Engelhardt-Wendt E, Weyermann B | 2008 | UNHCR | Grey | Global humanitarian settings | Increasing women’s leadership and empowerment. |
| 14 | Stephanie Parker | 2015 |  | Published | Syrian refugees | Not applicable |
| 15 | Partnership for Maternal, Newborn & Child Health | 2019 | Partnership for Maternal, Newborn & Child Health | Grey | Global humanitarian settings | Not applicable |
| 16 | Benjamin Black, Paul Bouanchaud, Jenine Bignall, Emma Simpson, Manish Gupta | 2014 |  | Published | Global conflict settings | Not applicable |
| 17 | Leontine Alkema, Elena Broaddus, Doris Chou, Daniel Hogan, Colin Mathers, Ann-Beth Moller, Lale Say, Sanqian Zhang | 2015 |  | Published | Global | Not applicable |
| 18 | UNSC | 2000 | UNSC | Grey | Global conflict settings | Not applicable |
| 19 | Kristen Meagher | 2019 | R4HC-MENA | Grey | None specified | Women leaders in global health. |
| 20 | Global Health 50/50 | 2020 | Global Health 50/50 | Grey | Global | Quantitative data on women leaders in global health organisations. |
| 21 | Kaamil Ahmed | 2020 | The Guardian | Grey | Global | Quantitative data on women leaders in global health organisations. |
| 22 | Ayoade Olatunbosun-Alakija | 2019 |  | Grey | None specified | Advancing women’s leadership in conflict and humanitarian settings. |
| 23 | Fathiah Zakham, Katia Jaton | 2019 |  | Published | Yemen | Advancing women in science. |
| 24 | Council of Europe | 2011 | Council of Europe | Grey | None specified | Not applicable |
| 25 | Council of Europe | 2019 | Council of Europe | Grey | None specified | Not applicable |
| 26 | Helen Clark | 2019 | The Global Institute for Women's Leadership, King's College London | Grey | None specified | Reframing gender equality, organisational policies and structural change to support women leaders. |
| 27 | Interagency Standing Committee | 2018 | Interagency Standing Committee | Grey | None specified | Not applicable |
| 28 | World Health Organisation | 2011 | World Health Organisation | Grey | Global | Gender integration at the World Health Organisation; enhance leadership skills of female staff. |
| 29 | Conflict and Health |  |  | Not applicable | Not applicable | Not applicable |
| 30 | Barbara Mazur | 2010 |  | Published | None specified | Inclusion of women to support diversity in the workplace. |
| 31 | Hannah Blackney, Seeta Giri, Pip Henty, Kate Sutton | 2019 | Humanitarian Advisory Group | Grey | Global humanitarian settings | Data of women in humanitarian leadership. |
| 32 | Russell Mannion, Hue Davies | 2018 |  | Published | UK | Not applicable |
| 33 | Paul Knox Clarke | 2013 | ALNAP | Grey | Global humanitarian settings | Organisational structure and underrepresentation of women in humanitarian organisations. |
| 34 | Ricardo Fal-Dutra Santos | 2019 | ICRC | Grey | None specified | Gender transformative action. |
| 35 | Devex | 2019 | Devex | Grey | Global humanitarian settings | Barriers to increased female leadership in humanitarian settings. |
| 36 | Jane Ritchie, Liz Spencer | 2002 |  | Published | None specified | Not applicable |
| 37 | Virginia Braun, Victoria Clarke | 2006 |  | Published | None specified | Not applicable |
| 38 | Ayla Black, Pip Henty, Kate Sutton | 2017 | Humanitarian Advisory Group | Grey | Global humanitarian settings | Humanitarian leadership gender gap and related barriers. |
| 39 | Khalid Arar, Izhar Oplatka | 2016 |  | Published | Middle East | Research on women leaders in education. |
| 40 | Kristen Meagher, Neha Singh | 2020 |  | Published | Global humanitarian and conflict settings | Barriers and opportunities for women’s leadership in conflict and humanitarian settings. |
| 41 | Alice Eagly, Mary Johannesen-Schmidt, Marloes Van Engen | 2003 |  | Published | None specified | Gendered leadership styles. |
| 42 | UNOCHA | 2019 | UNOCHA | Grey | Global humanitarian settings | Perspectives of women working in the humanitarian field. |
| 43 | Alejandra Pieda, Sophie Purdue | 2019 |  | Published | Asia and the Pacific | Feminist design in research projects to support women’s leadership. |
| 44 | Mishal Sameer Khan, Fatim Lakha,  Melisa Mei Jin Tan, Shweta Rajkumar Singh, Rina Yu Chin Quek, Emeline Han, See Mieng Tan, Victoria Haldane, Montserrat Gea-Sánchez, Helena Legido-Quigley | 2019 |  | Published | UK and US | Leadership schemes and support at global health universities. |
| 45 | Valentina Beghini, Umberto Cattaneo, Emanuela Pozzan, Mari Dahl Schlanbusch | 2019 | International Labour Office | Grey | Global | Barriers to women’s leadership in various contexts. |
| 46 | Ratna Sahay, Martin Cihak | 2018 | International Monetary Fund | Grey | None specified | Women’s leadership in the financial sector. |
| 47 | Larry Elliot | 2019 | The Guardian | Grey | None specified | Increasing women’s leadership to support the economy. |
| 48 | World Economic Forum | 2019 | World Economic Forum | Grey | Global | Creating policies to advance more women into leadership roles and diversify leadership across sectors. |
| 49 | Jyoti S Mathad , Lindsey K Reif, Grace Seo , Kathleen F Walsh, Margaret L McNairy, Myung Hee Lee, Adolfine Hokororo , Aarti Kinikar, Claudia T Riche, Marie M Deschamps, Sandy Nerette, Smita Nimkar, Neema Kayange, Hyasinta Jaka, Glory Joseph, Domenica Morona, Thandiwe Yvonne Peter, Nishi Suryavanshi, Daniel W Fitzgerald, Jennifer A Downs | 2019 |  | Published | US, Haiti, Tanzania | Women’s leadership in global health. |
| 50 | International Federation of Red Cross and Red Crescent Societies (IFRC) | 2018 | IFRC | Grey | Global | Increasing the percentage of women that hold leadership positions at the IFRC. |
| 51 | Sarah Hyde, Kate Hawkins | 2018 |  | Published | Cambodia | Promoting women’s leadership in the post-conflict health sector. |
| 52 | Paul Spiegel | 2017 |  | Published | Global humanitarian settings | Remake leadership in humanitarian settings. |
| 53 | Centre for Humanitarian Leadership | 2019 | Centre for Humanitarian Leadership | Grey | Global humanitarian settings | Having more women humanitarian leaders will help transform the humanitarian system. |
| 54 | Helen Lindley-Jones, Toral Pattni | 2018 | Humanitarian Practice Network | Grey | Global humanitarian settings | Increase the participation and leadership of women responders in humanitarian responses. |
| 55 | Brandon A Kohrt, Amit S Mistry, Nalini Anand, Blythe Beecroft, Iman Nuwayhid | 2019 |  | Published | Global humanitarian settings | Not applicable |
| 56 | Karen Longman, Jessica Daniels, Debbie Lamm Bray, Wendy Liddell | 2018 |  | Published | US | Organisational culture and women’s leadership in higher education |
| 57 | Roya Ayman, Karen Korabik | 2010 |  | Published | None specified | The influence of gender and culture on leadership. |
| 58 | Deborah O’Neil, Margaret Hopkins, Diana Bilimoria | 2008 |  | Published | None specified | Organisational culture and women’s leadership. |
| 59 | Charlotte M. Karam, Fida Afiouni | 2013 |  | Published | Middle East and North Africa | Barriers to women’s leadership in academia. |
| 60 | United Nations Economic and Social Commission for Western Asia | 2017 | United Nations Economic and Social Commission for Western Asia | Grey | Arab region | Women’s representation and leadership in politics. |
| 61 | Amr Eleraqi, Islam Salahuddin | 2018 | Al-Fanar Media | Grey | Arab region | Women’s leadership in higher education. |
| 62 | Valery Ridde, Christian Dagenais, Isabelle Daigneault | 2019 |  | Published | None specified | Sexual violence towards women in academic global health. |
| 63 | Lara Seigneur, Jose Chacon | 2017 | Centre for Humanitarian Leadership | Grey | Guatemala, El Salvador, Honduras and Nicaragua | Challenges women leaders in face in humanitarian settings, and how humanitarian trainings could better cater to leadership development. |
| 64 | Alison Barclay, Michelle Higelin, Melissa Bungcaras | 2016 | Action Aid | Grey | Philippines, Bangladesh, Ethiopia, occupied Palestinian territory, Nepal | Barriers and opportunities for women’s leadership in humanitarian responses. |
| 65 | Dyan Mazurana, Phoebe Donnelly | 2017 | Feinstein International Center | Grey | Global humanitarian settings | Sexual assault against humanitarian and development aid workers and organisational leadership. |
| 66 | Sonya Ruparel, Clare Bleasdale, Kathleen O’Brien | 2017 | Action Aid | Grey | Global humanitarian settings | Encouraging more women leaders in humanitarian responses and its importance. |
| 67 | Gabriella Swerling | 2019 | The Telegraph | Grey | Global conflict settings | Not applicable |
| 68 | Ben Quinn | 2018 | The Guardian | Grey | Global humanitarian settings | Not applicable |
| 69 | UK House of Commons | 2018 | UK House of Commons | Grey | Global humanitarian settings | Zero tolerance policies and organisational culture. |
| 70 | Christopher Hope | 2018 | The Telegraph | Grey | UK | Not applicable |
| 71 | Emma Smith, Rebecca Root | 2019 | Devex | Grey | Global humanitarian settings | Increasing women’s leadership in global development and zero tolerance policies. |
| 72 | Deloitte | 2019 | Deloitte | Grey | None specified | Organisational culture. |
| 73 | Renate Ysseldyk, Katharine H. Greenaway, Elena Hassinger, Sarah Zutrauen, Jana Lintz, Maya P. Bhatia, Margaret Frye, Else Starkenburg, Vera Tai | 2019 |  | Published | North America, Europe | Barriers to women’s leadership in academia. |
| 74 | Jessica K Paulus, Karen M Switkowski, Geneve M Allison, Molly Connors, Rachel J Buchsbaum , Karen M Freund , Deborah Blazey-Martin | 2016 |  | Published | US | Barriers to women’s leadership in academia. |
| 75 | Laurie Garrett | 2018 |  | Published | Global | Barriers to women’s leadership in medical practitioners and researchers. |
| 76 | Zohray Talib, Katherine States Burke, Michelle Barry | 2017 |  | Published | None specified | Women leaders in global health conference. |
| 77 | Matthew Hutson | 2018 |  | Published | US | Leadership styles. |
| 78 | Alice Eagly | 2007 |  | Published | US | Leadership styles and the challenge of female leaders to succeed in male-dominated leadership roles. |
| 79 | Aula Abbara | 2019 | Imperial College London | Grey | Middle East | Disparities in the experiences of female health researchers. |
| 80 | Meghna Basu  Thakur, Priscilla Paul | 2017 |  | Published | US | Sexual harassment in academic institutions. |
| 81 | Amir Karami, Cynthia Nicole White, Kayla Ford, Suzanne Swan, Melek Yildiz Spinel | 2019 |  | Published | India | Sexual harassment in academic institutions. |
| 82 | Stephen J. Aguilar, Clare Baek | 2020 |  | Published | US | Sexual harassment in academic institutions. |
| 83 | Abdul Hadi | 2018 |  | Published | Pakistan | Sexual harassment in academic institutions. |
| 84 | OECD | 2019 | OECD | Grey | Development Assistance Committee (DAC) members | Aid focused on gender equality and women’s empowerment. |
| 85 | Isabela Vera, Francesca Sanders | 2019 | Seek Development | Grey | Development Assistance Committee (DAC) members | Official development assisting funding gender equality and women’s empowerment. |
| 86 | Diana Koester, Emily Esplen, Karen Barnes Robinson, Clare Castillejo, Tam O'Neil | 2016 |  | Published | Conflict and fragile settings | Top-level leadership as a key enabler in improving how gender is integrated into donor programmes in fragile and conflict-affected contexts. |
| 87 | Maria Al-Abdeh, Champa Patel | 2019 |  | Published | Syrian women in Syria and neighbouring countries | Transformative approaches to women’s leadership. |
| 88 | Michelle Lokot | 2019 |  | Published | Syrian refugees in Jordan and humanitarian workers | Feminist research design. |
| 89 | Namalie Jayasinghe, Momotaz Khatun, Moses Okwii | 2020 | Oxfam | Grey | Bangladesh and South Sudan | Barriers to women’s organisations and local women’s leadership. |
| 90 | Nassim El Achi, Andreas Papamichail, Antony Rizk, Helen Lindsa, Marilyne Menassa, Rima Abdul-Khalek | 2019 |  | Published | Middle East and North Africa | Gender equity. |
| 91 | Katerina Dalacoura | 2019 | MENARA | Grey | Middle East and North Africa | Mapping women’s organisations and women’s empowerment during the Arab Spring. |
| 92 | Selma Scheewe | 2017 | Red R | Grey | Jordan | Empowering female leaders in the humanitarian sector, leadership styles, women working in traditionally male dominated professions in NGOs. |
| 93 | The Kuwait Foundation for the Advancement of Sciences | 2017 | The Kuwait Foundation for the Advancement of Sciences | Grey | Kuwait | Supporting aspiring women leaders in science. |
| 94 | Rebecca Root, Emma Smith | 2019 | Devex | Grey | Global humanitarian settings | Strategies for increasing female leadership in the humanitarian and development sector. |
| 95 | Kristen Meagher | 2019 | R4HC-MENA | Grey | Palestine | Opportunities for women’s leadership in health and conflict through academia and partnerships. |
| 96 | Jocalyn Clark, Elizabeth Zuccala, Richard Horton | 2017 |  | Published | None specified | Barriers to women’s leadership: disadvantage, discrimination, and sexism. |
| 97 | Nina Schwalbe, Jennifer Fearon | 2018 |  | Published | None specified | Gender bias in global health publications. |
| 98 | Gemma Bowsher, Andreas Papamichail, Nassim El Achi, Abdulkarim Ekzayez, Bayard Roberts, Richard Sullivan, Preeti Patel | 2019 |  | Published | Global conflict settings | Gender as an important locus of inequity in health capacity research and health systems. |
| 99 | Fogarty International Centre |  | National Institutes of Health | Grey | Global humanitarian settings | Not applicable |
| 100 | Natalie Boychuck | 2018 | Grand Challenges Canada | Grey | Syria and global humanitarian settings | Improving the ability of humanitarian programme to engage with and be led by women. |
| 101 | Preeti Patel (personal correspondence) | 2018 |  | Not applicable | Global conflict settings | Ratios of female vs. male principal investigators in conflict/health research studies. |
| 102 | International Agency Standing Committee | 2019 | International Agency Standing Committee | Grey | Global humanitarian settings | Mapping male and female humanitarian coordinators. |
| 103 | UN Women | 2015 | UN Women | Grey | Global humanitarian settings | Removing structural barriers to gender equality and women’s empowerment. |
| 104 | Beniamino Cislaghi,Elaine K Denny EK, Mady Cissé, Penda Gueye, Binita Shrestha, Prabin Nanicha Shrestha, Gemma Ferguson, Claire Hughes, Cari Jo Clark | 2019 |  | Published | Mali, Nigeria, Nepal | Changing social norms to empower women. |
| 105 | Martina Angela Caretta, Danielle Drozdzewski, Johanna Carolina Jokinen, Emily Falconer | 2017 |  | Published | Global North | Career progression for women in academia. |
| 106 | Marina Ranga, Namrata Gupta, Henry Etzkowitz | 2012 | Deutsche Forschungsgemeinschaft | Grey | Europe | Lack of women in top leadership or management positions of public national research funding organisations. |
| 107 | Katherine a. H. Graham, Allan M. Maslove | 2017-2018 | Carleton University | Grey | Canada and foreign aid. | Insufficient donor funding to support women’s empowerment. |
| 108 | OECD | 2017 | OECD | Grey | Fragile and Conflict-Affected Situations | Donor funding to support women’s empowerment and gender equality. |
| 109 | Nassim El Achi, Gladys Honein-Abouhaidar, Anthony Rizk, Elsa Kobeissi, Andreas Papamichail, Kristen Meagher, Preeti Patel, Ghassan Abu-Sittah | 2020 |  | Published | Lebanon | High representation of women in health research. |
| 110 | Alice H. Eagly, Linda L. Carli | 2003 |  | Published | None specified | Leadership styles and increasing number of women into leadership in elite executive roles. |
| 111 | Diana O’Brien, Johanna Rickne | 2019 |  | Published | Sweden | Gender quotas and women's Political Leadership. |
| 112 | Zahra Zeinali, Kui Muraya, Veloshnee Govender, Sasse Molyneux, Rosemary Morgan. | 2019 |  | Published | None specified | Intersectionality in support of diverse global health leadership |
| 113 | Sara E Davies Sophie Harman, Rashida Manjoo, Maria Tanyag | 2019 |  | Published | Global health | Gender diversity, intersectionality, hidden burden of care and feminist methodology. |
| 114 | Sharon Mavin, Gina Grandy, Jannine Williams | 2014 |  | Published | UK | Intra-gender relations. |
